# Clinical and Environmental Vibrio cholerae Non-O1, Non-O139 Strains from Australia Have Similar Virulence and Antimicrobial Resistance Gene Profiles

**DOI:** 10.1128/spectrum.02631-22

**Published:** 2023-01-23

**Authors:** Murari Bhandari, Irani U. Rathnayake, Flavia Huygens, Amy V. Jennison

**Affiliations:** a Centre for Immunology and Infection Control, Queensland University of Technology, Brisbane, Queensland, Australia; b Public Health Microbiology, Forensic and Scientific Services, Queensland Department of Health, Brisbane, Queensland, Australia; Suranaree University of Technology

**Keywords:** *Vibrio cholerae*, antimicrobial activity, clinical strains, environmental strains, gene sequencing, virulence factors

## Abstract

Cholera caused by pathogenic Vibrio cholerae is still considered one of the major health problems in developing countries including those in Asia and Africa. Australia is known to have unique V. cholerae strains in Queensland waterways, resulting in sporadic cholera-like disease being reported in Queensland each year. We conducted virulence and antimicrobial genetic characterization of O1 and non-O1, non-O139 V. cholerae (NOVC) strains (1983 to 2020) from Queensland with clinical significance and compared these to environmental strains that were collected as part of a V. cholerae monitoring project in 2012 of Queensland waterways. In this study, 87 V. cholerae strains were analyzed where O1 (*n* = 5) and NOVC (*n* = 54) strains from Queensland and international travel-associated NOVC (*n* = 2) (61 in total) strains were sequenced, characterized, and compared with seven previously sequenced O1 strains and 18 other publicly available NOVC strains from Australia and overseas to visualize the genetic context among them. Of the 61 strains, three clinical and environmental NOVC serogroup strains had cholera toxin-producing genes, namely, the CTX phage (identified in previous outbreaks) and the complete *Vibrio* pathogenicity island 1. Phylogenetic analysis based on core genome analysis showed more than 10 distinct clusters and interrelatedness between clinical and environmental V. cholerae strains from Australia. Moreover, 30 (55%) NOVC strains had the cholix toxin gene (*chxA*) while only 11 (20%) strains had the *mshA* gene. In addition, 18 (34%) NOVC strains from Australia had the type three secretion system and discrete expression of type six secretion system genes. Interestingly, four NOVC strains from Australia and one NOVC strain from Indonesia had *int*SXT, a mobile genetic element. Several strains were found to have beta-lactamase (*bla*_CARB-9_) and chloramphenicol acetyltransferase (*catB9*) genes. Our study suggests that Queensland waterways can harbor highly divergent V. cholerae strains and serve as a reservoir for various V. cholerae-associated virulence genes which could be shared among O1 and NOVC V. cholerae strains via mobile genetic elements or horizontal gene transfer.

**IMPORTANCE** Australia has its own V. cholerae strains, both toxigenic and nontoxigenic, that are associated with cholera disease. This study aimed to characterize a collection of clinical and environmental NOVC strains from Australia to understand their virulence and antimicrobial resistance profile and to place strains from Australia in the genetic context of international strains. The findings from this study suggest the toxigenic V. cholerae strains in the Queensland River water system are of public health concern. Therefore, ongoing monitoring and genomic characterization of V. cholerae strains from the Queensland environment are important and would assist public health departments to track the source of cholera infection early and implement prevention strategies for future outbreaks. Understanding the genomics of V. cholerae could also inform the natural ecology and evolution of this bacterium in natural environments.

## INTRODUCTION

Vibrio cholerae is a Gram-negative bacterium that can survive in aquatic ecosystems and can cause the life-threatening diarrheal disease cholera when transmitted to the host. To date, seven cholera pandemics have been recorded and are ongoing due to toxigenic O1 V. cholerae (1). The O1 and O139 serogroups are responsible for millions of death each year in underdeveloped and developing countries (2, 3). During evolution, V. cholerae acquired virulence genes, genomic islands, and mobile genetic elements that enabled this bacterium to become more virulent and resistant to antibiotics (4, 5). The cholera toxin (CTX) and the toxin-coregulated pilus (TCP) are the major virulence factors of the toxigenic O1 and O139 strains and are encoded on mobile genomic regions as filamentous phage CTXΦ (6, 7) and *Vibrio* pathogenicity island VPI-I, respectively (8, 9).

To date, based on the expression of the surface-expressed O antigen in V. cholerae strains, more than 200 serogroups have been classified. V. cholerae strains that are not expressing O1 and O139 antigens are broadly classified as NOVC strains (10). Globally, these NOVC strains are associated with moderate to severe gastroenteritis and extraintestinal infections such as wound and soft tissue infections, ear infections, or bacteremia (11–14). Initially, most of the NOVC strains were reported as nontoxigenic due to the lack of toxigenic CTX- and TCP-encoding genes and have been poorly studied worldwide (15).

However, in recent years several studies reported NOVC strains with toxigenic genes and additional mobile genetic elements that confer resistance to multiple antibiotics (16–19). In addition in NOVC strains, accessory virulence factors such as a transcriptional activator for toxin-related genes including *ctxAB* (*toxR*), mannose-sensitive hemagglutinin pilus (*mshA*), different hemolysins (*hlyA*), repeats in toxin (RTX) toxin clusters, outer membrane proteins (*ompU*), cholix toxin (*chxA*), heat-stable enterotoxin (*stn*), flagellum-associated cytotoxin (*makA*), and glucose metabolism (*als*), may contribute directly or in a synergistic way to the infection process leading to diarrheal illness (20–24). In *in vivo* studies, the type three and six secretion systems (TTSS and T6SS, respectively) play a crucial role in colonization and cause diarrheal disease caused by V. cholerae in animal models (25–27). Among NOVC strains, the distribution and the broad diversity of accessory virulence factors are common globally compared to toxigenic O1 and O139 strains (28).

Climate change and genetic epidemiology of V. cholerae are a topic of concern at present (1, 29). Of the different adaptational strategies that bacterial species including V. cholerae employ to survive in the changing environment, one of them is genetic variation by horizontal gene transfer or positional mutations. Serogroup switching and transfer of mobile genetic elements among different serogroups of V. cholerae strains via horizontal gene transfer and disease occurrence are of keen interest currently. Clinical NOVC strains that contain genes pathogenic to humans despite not being O1/O139 V. cholerae strains are of public health concern. Recently, NOVC clinical V. cholerae strains exhibited genotypic profiles similar to the O1 El Tor Haiti (CT) variant in a 5-month-old baby in India (19). Furthermore, several NOVC clinical and environmental isolates carrying mobile genetic elements and thereby acquiring resistance to multiple antibiotics are of global concern because, in general, SXT genetic elements play a role in the evolution of the isolate (19, 30, 31).

Whole-genome sequencing (WGS) is a powerful tool and a widely used approach for comprehensive evaluation of phylogenetic relations based on single nucleotide polymorphisms (SNPs) to track the epidemiology of V. cholerae globally. In recent years, WGS is becoming the method of choice in research settings for monitoring pathogens, including V. cholerae, for diagnostic purposes where sequence data can be utilized for (i) development of novel antibiotics, (ii) detection of the emergence of antibiotic resistance, (iii) outbreak identification, (iv) pathogen surveillance, (v) development of novel diagnostic tests, (vi) implementation of direct infection control measures, and (vii) evolutionary studies (1).

In some regions of Australia, sporadic clinical cases related to NOVC infections have been reported from 1983 to the present time with or without travel history (14, 32, 33). In addition, the NOVC bacterium arises in marine, estuarine, and even freshwater locations under favorable environmental conditions (temperature, chitin availability, etc.), which makes studying Queensland (QLD) waterways particularly important. All clinical and environmental NOVC strains isolated in Queensland from human and animal infections are collected and stored at the public health microbiology (PHM) laboratory, Queensland Health Department. However, until now, no molecular or genomic studies of Queensland NOVC strains have been reported, particularly with a focus on virulence and antimicrobial resistance traits. This study has extensively investigated the genotypic features of clinical and environmental NOVC strains from Australia as having similar virulence potential. Interestingly, in this study we confirmed the presence of *ctxB* genotype 2, CTX phage region, among environmental NOVC strains from Australia similar to toxigenic Queensland outbreak-related O1 El Tor V. cholerae clinical and environmental strains from our previous study (34). Thus, based on our characterization of these strains QLD waterways harbor genetically diverse V. cholerae strains, which may be due to the mechanism of horizontal gene transfer and the bipartite architecture of V. cholerae’s genome.

## RESULTS

### SNP-based phylogenetic analysis.

**FIG 1:**
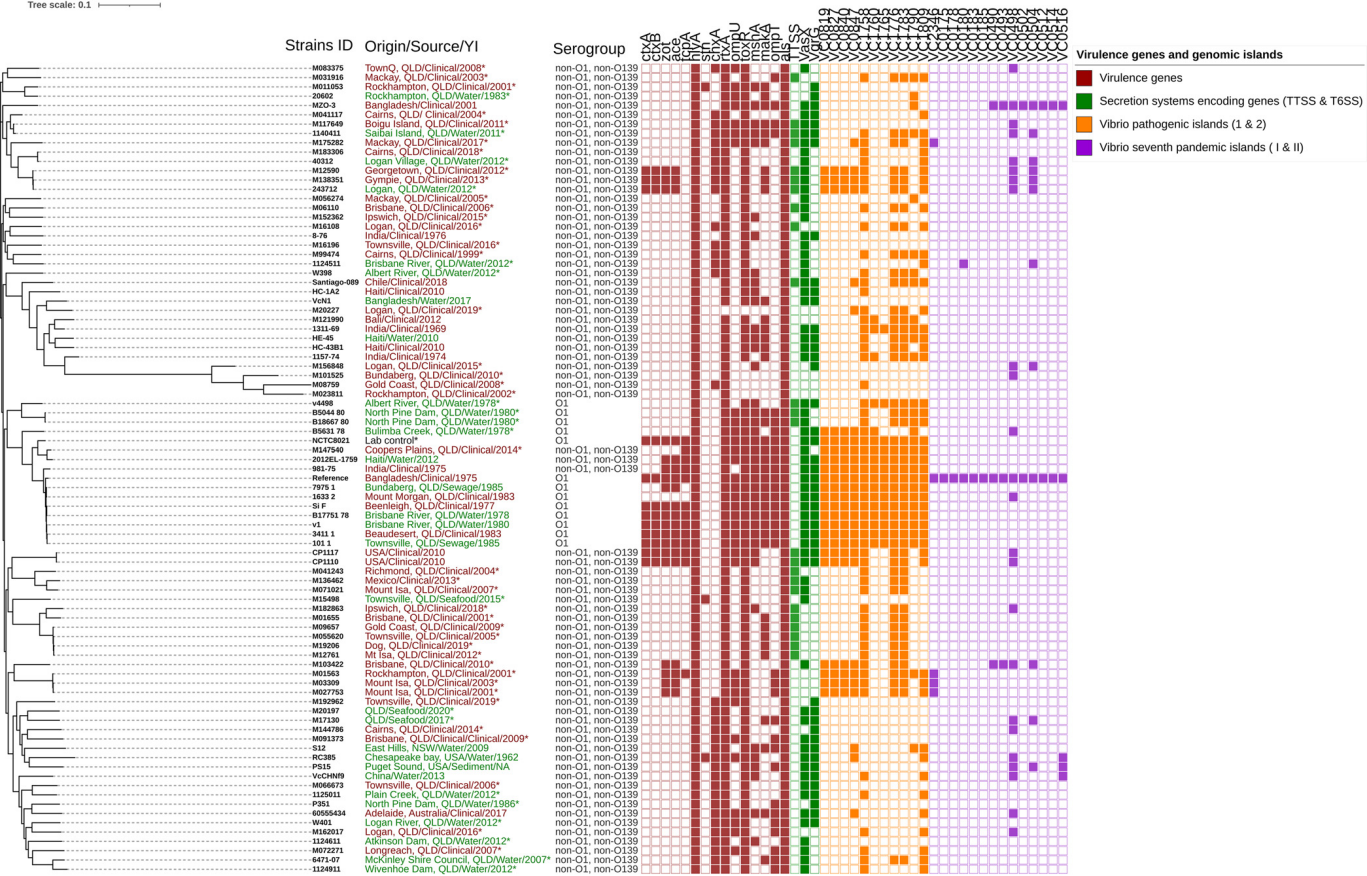
Maximum likelihood tree showing phylogenetic relationships between 87 clinical (human and animal infections) and environmental V. cholerae strains: O1 (*n* = 13), NOVC (*n* = 56), and publicly available (*n* = 18) V. cholerae strains with origin of isolates, source, year of collection (YI), serogroup and virulence genes, *Vibrio* pathogenicity islands 1 and 2 (VPI-1 and -2), and *Vibrio* seventh-pandemic islands I and II (VSP-I and -II). Gene profiles were generated using iTOL (https://itol.embl.de/) and represented as a colored box for presence and a white box for absence. Strains sequenced in this study were noted with an asterisk (*). Description of origin, source, and year of isolation in red represents clinical samples, and that in green represents environmental samples.

Among the 87 V. cholerae clinical and environmental strains used in this study (Fig. 1; see also Data Set S1 in the supplemental material), serotype O1 strains (*n* = 5), NOVC strains (clinical = 39, environmental = 15) from Australia, and international travel-associated NOVC strains (*n* = 2) (61 in total) were sequenced, characterized, and compared to seven previously sequenced strains representing a different disease cluster (34), reference (N16961) O1 strains, and 18 additional publicly available Australian and international NOVC strains. As shown in the maximum likelihood phylogenetic tree (Fig. 1), the strains clustered into several distinct groups and subgroups. Based on this SNP analysis, overall SNP differences among NOVC strains were ~30,000 SNP differences except for four outgrouped clinical strains (M08759, M023811, M101525, and M156848) with ~80,000 SNP differences. Interestingly, several clinical non-O1 and non-O139 strains are closely related to environmental strains, and notably, clinical strains from different Queensland regions from the same or different isolation times clustered closely (Fig. 1). Clinical NOVC strains (M138351 and M12590) isolated in 2013 and 2012 from the Gympie and Georgetown regions showed only nine and 16 SNP differences compared to the Albert River, Logan region, water isolate (243712) (Fig. 1), respectively. Some of our local strains (M20227, M071021, and 1124511) belonged to a monophyletic clade that includes a water strain from Bangladesh and clinical strains from Mexico and India. All the O1 V. cholerae strains from this study and selected O1 strains from previous studies clustered into the same monophyletic clade with low SNP differences and distinct distance from other NOVC strains except strain M147540 (Fig. 1). Overall, despite the divergence of strains in the cluster, most of the local environmental non-O1 and non-O139 isolates were interrelated to clinical cases even though there is a difference in the year of isolation.

### Genotypic characterization of NOVC strains.

**FIG 2:**
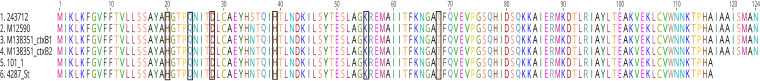
Multiple-sequence alignment of CtxB amino acid sequences from three NOVC strains (243712, M12590, and M138351) compared to two previously sequenced O1 serogroup strains from Australia (101_1 and 4287_St). Black-edge rectangles represent the position of *ctxB* genotype 2-distinguishing amino acid sequences.

Of the 56 sequenced strains from Australia and travel-associated NOVC strains, only three strains harbored the CT-producing *ctxAB* genes and are hence classified as toxigenic strains. For the first time in Queensland cholera history, *ctxB* genotype 2 was determined to be present in one environmental and two clinical NOVC serogroup strains as shown in Fig. 2 and Table 1. Moreover, only a few NOVC strains from Australia had mixed genotypes with respect to repetitive sequence transcriptional repressor (*rstR*) of classical type and/or El Tor type: one with toxin-coregulated pilus *tcpA* of classical type and 48 (90%) lacking biotype-specific genotypes. It is interesting to note the presence of classical and El Tor *rstR* and *ctxB* gene sequences together in one of the environmental NOVC strains from Australia that was isolated in 2012 from Logan’s Albert River, while other toxigenic or nontoxigenic strains were either/or (classical or El Tor) positive for the *rstR* gene as shown in Table 1. Surprisingly, while extracting *ctxB* genes to translate for multiple sequence alignment and genotyping of toxigenic NOVC strains, two *ctxB* gene regions in one of the strains (M138351) were detected, and translated protein sequences are compared as shown in Fig. 2.

**TABLE 1:** Diverse genotypes of NOVC strains investigated in this study

Country of origin	Biotype-specific genotype^*a*^							
	*ctxB*-Aus	*ctxB*-CC	*ctxB*-ET	*rstR*-ET	*rstR*-CC	*tcpA*-CC	*tcpA*-ET	*ctxB* genotype
Australia (*n* = 1) (243712)	+			+	+			2
Australia (*n* = 2) (M027753 and M03309)				+				
Australia (*n* = 2) (M12590 and M138351)	+				+			2
Australia (*n* = 2) (M01563 and M147540)						+		NA^*b*^
India (*n* = 1) (981-75)					+		+	

a +, presence; Aus, Australia; ET, El Tor; CC, classical.

b NA, not applicable.

As shown in Fig. 1, all the sequenced NOVC strains showed diverse virulence profiles. In this study, 53 (95%) NOVC strains sequenced were nontoxigenic and three toxigenic strains notably had other virulence genes such as *chxA*, *stn*, *rtxA*, *ompU*, *ompT*, *toxR*, *mshA*, *makA*, and *als* and type three secretion system and type six secretion system genes as well. All 54 clinical and environmental NOVC strains from Australia contained hemolysin and multifunctional autoprocessing repeats-in-toxin genes (*hlyA* and *rtxA*), whereas the cholera toxin transcriptional activator *toxR* gene was absent in only five clinical NOVC strains (Fig. 1). This means 100% of environmental strains contained either separate or a combination of *rtxA*, *hlyA*, and *toxR* genes, signifying the pathogenic potential of these environmental NOVC strains.

Three toxigenic NOVC strains and four NOVC strains with a truncated CTX phage region and *zot* and *ace* genes also contained an entire VPI-1 and a partial VPI-2 region. None of the V. cholerae O1 and NOVC strains harbored complete versions of both VSP-I and VSP-II. However, it was interesting that three clinical NOVC strains (M01563, M027753, and M03309) isolated from the Townsville and Mount Isa regions had the VC2346 gene sequence that differentiates seventh-pandemic strains from classical strains. Among the 55 V. cholerae NOVC strains, 30 (55%) had the cholix toxin gene (*chxA*) while only 11 strains had the *mshA* gene. In addition, 18 NOVC strains had the type three secretion system, whereas the presence of type six secretion system genes *vasX* and *vgrG* was randomly distributed. V. cholerae O1 strains sequenced in this study were nontoxigenic, lacking the CTX phage sequence while containing accessory virulence genes and secretion systems as shown in Fig. 1. Overall, it was interesting that there were no distinct virulence profiles for clinical isolates only, but similar virulence gene profiles were detected in clinical and environmental NOVC strains in Queensland.

### Presence of antimicrobial resistance gene profiles, class 1 integrons, and mobile genetic SXT element.

**FIG 3:**
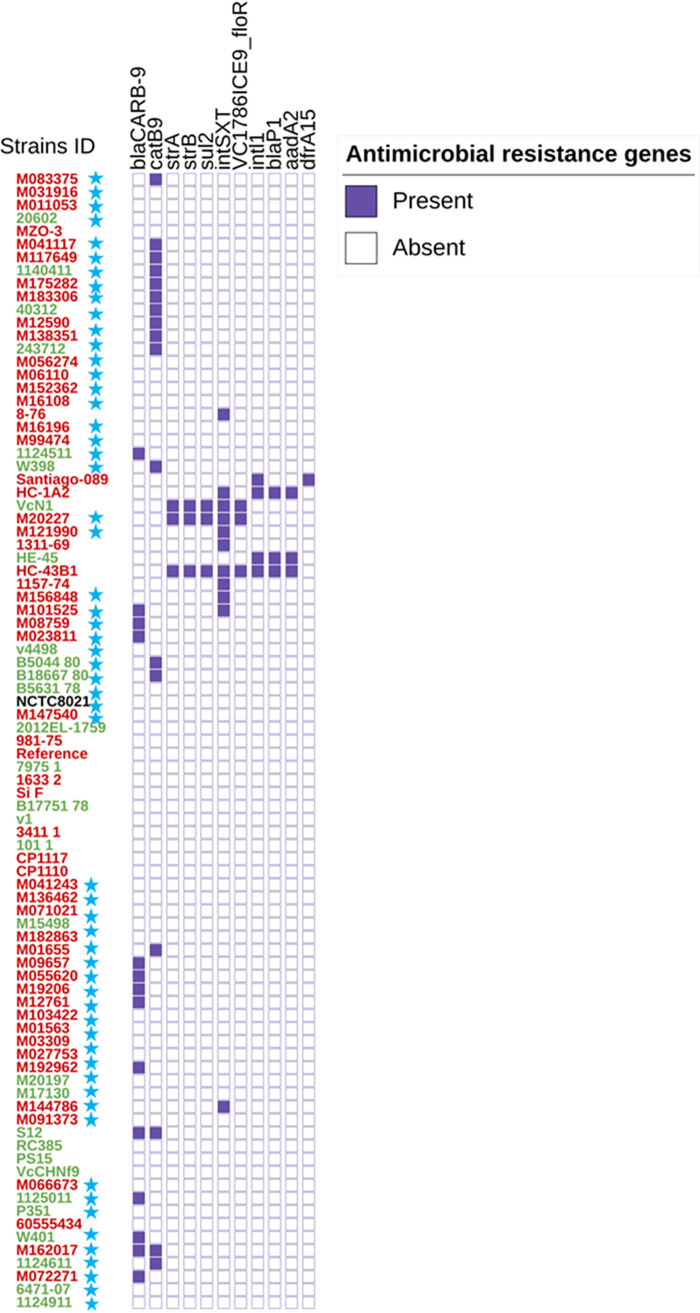
Presence or absence display annotation on iTOL for the clinical and environmental O1 and NOVC strains from Australia and other countries, their antimicrobial resistance genes, and mobile genetic element profile. The source of clinical V. cholerae strains is highlighted in red and that of environmental strains in green. Blue stars represent the strains sequenced in this study.

*In silico* analysis of all 87 sequences was performed to extract antimicrobial resistance gene profiles, class 1 integrons, and mobile genetic elements using the CholeraFinder tool. All the local aquatic and clinical isolates from Australia exhibited the same profile with no SNP differences among antimicrobial resistance-associated genes DNA gyrase subunit A, DNA topoisomerase IV subunit A, and DNA topoisomerase IV subunit B (*gyrA*, *parC*, and *parE*). Of the 56 NOVC strains, 17 (30%) had the chloramphenicol acetyltransferase gene *catB9*, whereas 13 (23%) had the *bla*_CARB-9_ gene. Surprisingly, four NOVC strains from Australia and one NOVC strain from Indonesia (clinical and environmental strains) had the *int*SXT mobile genetic element. Also, one of the strains from Logan Hospital (clinical strain M20227 isolated in 2019) carried genes that encode resistance to chloramphenicol (encoded by the VC1786ICE9_*floR* gene), sulfamethoxazole (*sul2*), and streptomycin (*strA* and *strB*) gene sequences as shown in Fig. 3.

### CTX phage analysis of NOVC and O1 V. cholerae strains from Australia.

**FIG 4:**
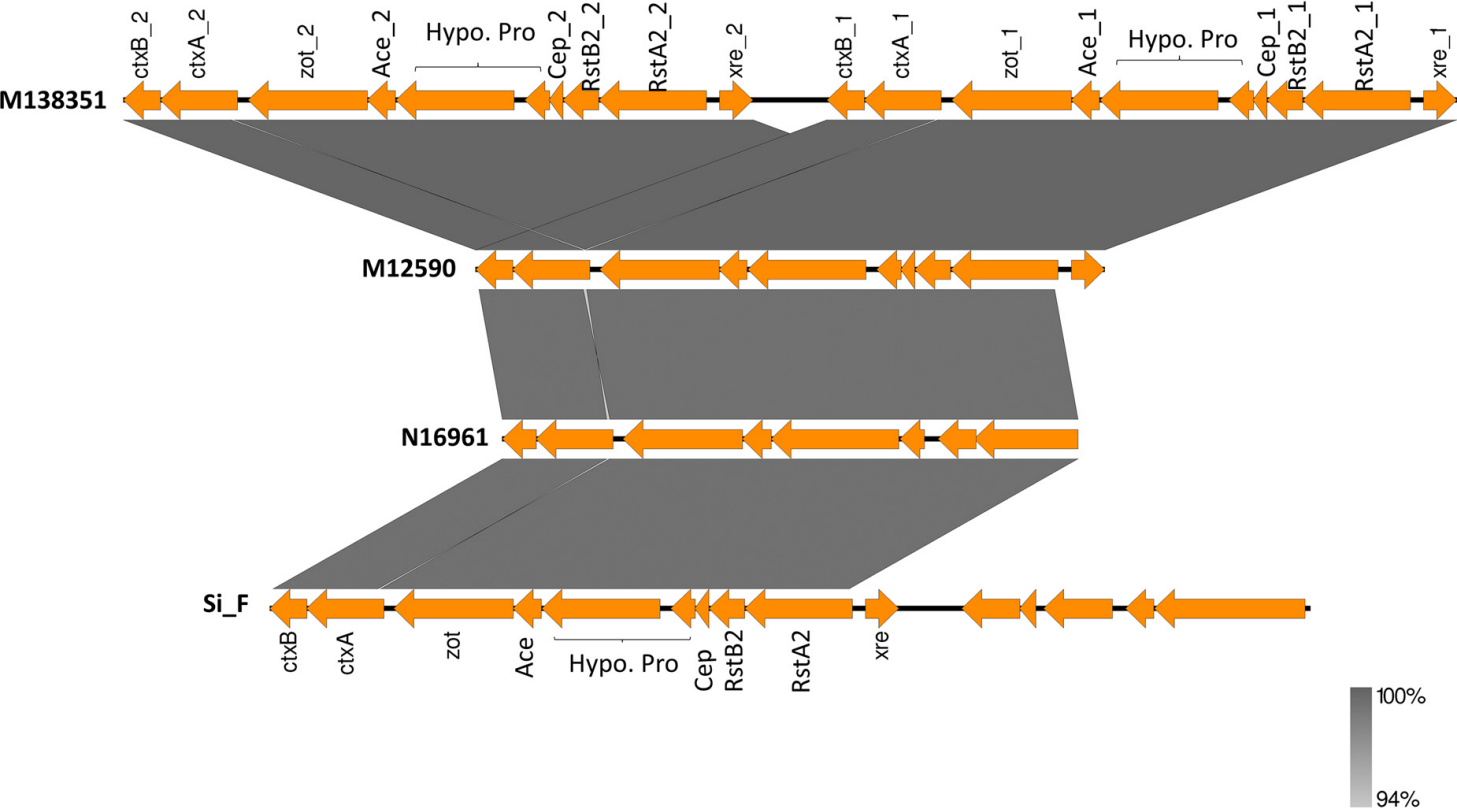
Sequence comparison of CTX phage regions integrated into the chromosome of O1 and NOVC strains. M138351 and M12590 CTX phage sequences are from NOVC strains sequenced in this study and compared to the CTX phage sequence of O1 El Tor strain Si_F from Australia and strain N16961 from Bangladesh. The intensity of color represents the level of identity as shown in parentheses.

The presence of cholera toxin genes in some NOVC strains from Australia was novel and is of public health concern. Thus, to elucidate the composition of the cholera toxin-producing CTX phage of NOVC strains and to compare their genetic similarity with other toxigenic CTX phages of the O1 El Tor strain from Australia and seventh-pandemic reference strain N16961, CTX phage sequences were extracted using PHASTER (https://phaster.ca/), and Easyfig was used for visualization of genomic regions. As a result, interestingly, two very similar CTX phage regions were identified in one of the clinical strains (M138351) as shown in Fig. 4. A similar genetic composition was found for the CTX phage regions in NOVC and reference O1 strains from Australia and Bangladesh, except for some variations in the *ctxA* gene of O1 and NOVC strains from Australia compared to N16961 (indicated by gaps in Fig. 4).

Table 2 provides the diversity in the locations of CTX prophage in the chromosome and the copy number and source among O1 El Tor strains from Australia and international V. cholerae O1 and NOVC strains.

**TABLE 2:** V. cholerae strains with chromosomal location of CTX phage region and copy number

Strain	Serotype (biotype)	Source	Origin	Chromosomal location of CTX prophage (copy no.)	Reference(s)
O395	O1 (classical)	Clinical	India	Large (1), small (1)	61
N16961	O1 (El Tor)	Clinical	Bangladesh	Large (1)	62
VC44	O1 (El Tor)	Clinical	India	Small (2)	63
Si_F	O1 (El Tor)	Clinical	Australia	1	34
v1	O1 (El Tor)	Environment	Australia	1	34
VCE232	Non-O1, non-O139	Environment	India	Large (2),^*a*^ small (2)	64, 65
M12590	Non-O1, non-O139	Clinical	Australia	1	This study
M138351	Non-O1, non-O139	Clinical	Australia	2	This study
243712	Non-O1, non-O139	Environment	Australia	1	This study

a CTX prophages lack *ctxAB* genes.

## DISCUSSION

V. cholerae is a dynamic habitant of the environment that can survive under unfavorable environmental conditions in a dormant state for some time, being viable but not culturable, and can revert to its pathogenic potential upon favorable conditions (35, 36). Globally, V. cholerae is an extensively studied pathogen mainly isolated in underdeveloped and developing countries, with little understanding of the evolution and pathogenesis of NOVC strains from developed countries like Australia. Genetic diversity among NOVC strains increases the complexity in understanding their origin and phylogenetic relationships. In this study, core genome phylogenetic analysis was used to understand the possible source of human and animal infections caused by V. cholerae NOVC strains. Several clinical NOVC strains showed close relationships to environmental strains from different regions and isolated at different times. However, it is challenging to differentiate and correlate their relationship without associated metadata. One of the clinical nontoxigenic V. cholerae non-O1, non-O39 strains (M20227), isolated in 2019 from Australia, had a similar antimicrobial resistance gene profile with 18,107 SNP differences and is closely related to an environmental nontoxigenic NOVC strain (VcN1), isolated in 2017 from Bangladesh. Bangladesh is renowned for many cholera outbreaks, and its environment also acts as a reservoir for V. cholerae strains (37). It would be interesting to know if the genotypic and antimicrobial resistance (AMR) similarities between VcN1 and M20227 strains have evolved independently or whether these traits are linked to transmission, which is likely due to the highly accessible nature of travel across countries.

Evolution of V. cholerae is a complex and interesting process, and over time, it has been proposed that V. cholerae has acquired several toxins and virulence and antimicrobial resistance genes via several genomic islands and bacteriophages through horizontal gene transfer, subsequently sharing its genes with other strains (1). Interestingly, in this study it was found that an environmental NOVC strain isolated in 2012 with the classical *rstR* and El Tor *rstR* genes was different from other strains that have either the classical *rstR* or the El Tor *rstR* gene, indicating the possible fitness requirement of the bacterium to survive in the environment. This might also play a role in the evolution process of NOVC by transferring necessary elements among serogroups.

In a similar way, genotype 2 *ctxB*-containing CTX prophage was previously reported in outbreak-related V. cholerae O1 El Tor strains from Australia (34) and is also observed in clinical NOVC strains in this study. Notably, three of the toxigenic NOVC clinical and environmental strains, M12590, M138351, and 243712, had single or double copies of the genotype 2-containing CTX phage region as shown in Table 2. It is surprising and interesting to see that these three NOVC strains harbor CTX prophage genes (*ctxA*, *ctxB*, *zot*, and *ace*) without the *tcpA* gene. As *tcpA* serves as a receptor for CTX prophage, it is thus rare to acquire those virulence genes without the receptor genes. Similar to our study, environmental NOVC strains SCE188, SCE200, SCE201, and SCE223 from India isolated in 1997/1998 lacked *tcpA* genes while *ctxAB* genes were present (38). In a previous *in vitro* study, the possibility of CTX gene transfer from a toxigenic O1 strain to an environmental NOVC strain was confirmed with uncertainty on whether the entire genome was transferred or whether the phage genome was integrated with the recipient chromosome upon transfer (39). According to our analysis, the entire CTX phage region is transferred from a toxigenic O1 El Tor strain from Australia to environmental NOVC strains. It would be interesting to explore its pathogenic potential using *in vitro*/*in vivo* studies in the future.

The expression of multiple virulence factors in NOVC strains has been reported globally (14, 28, 40, 41, 42). In this study, 80% of NOVC strains carried accessory virulence toxin genes, colonization-aiding factors, and secretion systems that play a role in pathogenesis, such as *chxA*, *rtxA*, *hlyA*, *stn*, *mshA*, and the TTSS. Notably, two clinical and one environmental toxigenic NOVC strain also contained the cholix toxin gene in addition to CT-encoding genes. These virulence factors relate to human infection even in the absence of CT and *tcpA* genes. Moreover, in a recent study, TTSS conferred enhanced virulence in clinical NOVC strains (26). However, immunogenic response and loss of toxigenic genes after infection cannot be ruled out. Similar to this study, Schwartz et al. (2019) reported environmental and clinical V. cholerae non-O1 and non-O139 strains with similar virulence gene profiles, thereby providing evidence that environmental strains can be as virulent as clinical strains (43).

Initially, antibiotic resistance genes and mobile genetic elements conferring antibiotic resistance among epidemic and endemic V. cholerae O1 and O139 strains were reported globally with limited investigations of NOVC strains. Early studies on beta-lactam resistance acquisition that confers ampicillin resistance due to a plasmid-located beta-lactamase in V. cholerae from Asia were reported in 1977 (44). Several studies from the 1990s describe the prevalence of ampicillin resistance among environmental NOVC serogroup strains around the world (45–48). In our study, an interesting finding was that the *bla*_CARB-9_ gene, which confers resistance to beta-lactams, was found in 23% of environmental and clinical NOVC strains from Australia. Four beta-lactamases have been characterized in NOVC strains, namely, CARB-2 (PSE-1), CARB-6, CARB-7, and CARB-9. The *bla*_CARB_ genes have been reported broadly among different bacteria, possibly acquired through mobile genetic elements (49, 50). Recently, *bla*_CARB-9_ was detected in environmental NOVC strains from Argentina and Austria (17, 47). In this study, the chloramphenicol acetyltransferase gene *catB9* was found in environmental and clinical strains (30% and 25%, respectively), which has also been described in a recent study (17).

Initially, the SXT element was described in a V. cholerae O139 serogroup strain in India, thought to be sourced from Vibrio parahaemolyticus, and subsequently disseminated among O1 and NOVC serogroups very rapidly (16, 31, 51, 52). In this study, some of the Queensland clinical and environmental NOVC strains have the integrase gene of the SXT element, *int*SXT. Of note, of the four strains with the *int*SXT gene, one of the clinical NOVC strains also has *sul2*, *strA*, *strB*, and VC1786ICE9_*floR* genes and is closely related to an environmental strain from Bangladesh. Similar to our study, the presence of the *int*SXT SXT element, without any other resistance genes, has also been reported in studies from Bangladesh, India, Mexico, and Thailand (40, 41, 53).

Taken together, it is important to understand that the aquatic ecosystem provides a suitable platform under favorable environmental conditions for spreading antimicrobial resistance and virulence traits, particularly via bacteriophages through horizontal gene transfer between bacterial populations. Previously, a *recA*-mediated conversion of a nontoxigenic V. cholerae O1 strain into a toxigenic O1 strain was observed using the chitin-induced transformation pathway (39). Noting that chitin is a freely abundant natural carbon source in aquatic environments, these ecosystems potentially serve as reservoirs for the emergence of new pathogenic V. cholerae strains by transferring pathogenic gene clusters among V. cholerae strains (54, 55). The likely environmental source, including agricultural water use and ballast water imported from regions of endemicity across the world, containing strains with antimicrobial resistance genes and other mobile genetic elements, can lead to environmental-human transmission. These environmental V. cholerae strains can acquire new resistance genes and toxigenic mobile genetic elements that enable them to become more pathogenic and could cause human infections leading to outbreaks. Therefore, continuous monitoring of virulence and antimicrobial resistance genes in environmental V. cholerae strains, including O1 and NOVC serogroup strains, is important for the early detection and prevention of cholera outbreaks.

### Conclusion.

This study analyzed Queensland V. cholerae O1 and NOVC serogroup strains collected between 1983 and 2020. There is evidence to suggest that there is an abundance of pathogenic and antimicrobial-resistant V. cholerae strains in Queensland waterways; further research is needed to determine if these waterways are the cause of this. Moreover, the presence of similar virulence gene profiles and diverse antimicrobial resistance gene profiles among NOVC clinical and environmental strains from Australia is comparable to that in strains from regions where cholera is endemic, which is of concern. Although the occurrence of toxigenic V. cholerae in the natural environment is rare in a nonepidemic region like Australia, the discovery of Australian genotype 2 CTX phage in the Queensland River waterways, which could have been transferred from toxigenic previously reported V. cholerae strains from Queensland to environmental V. cholerae, demonstrates the feasibility of converting environmental nontoxigenic V. cholerae to toxigenic strains, which has the potential to cause human cholera disease. Similarly, additional mobile genetic elements can be acquired from other bacterial species in these aquatic ecosystems, enabling V. cholerae to become more virulent, more pathogenic, and more resistant to multiple antibiotics. Thus, it is crucial to monitor Queensland waterways regularly to identify the likely source of cholera outbreaks and to understand the evolution genetics of pathogenic and resistant strains of V. cholerae.

## MATERIALS AND METHODS

### Selection of strains, DNA extraction, and whole-genome sequencing.

NOVC strains included in this study were isolated from clinical or environmental samples (*n* = 61) and subjected to WGS, and their sources, locations of isolation, biotypes, sequence types, and year of isolation are outlined in Fig. 1. DNA was extracted from isolates grown overnight at 37°C on horse blood agar (Edwards Group Holdings, Australia), using the QIAsymphony DSP DNA minikit (Qiagen) according to the manufacturer’s protocol. DNA was prepared for sequencing using the Nextera XT kit (Illumina) and sequenced on the NextSeq500 using the NextSeq 500 Mid Output v2 kit (300 cycles) (Illumina) according to the manufacturer’s instructions. Sequence reads for the V. cholerae isolates were trimmed with Trimmomatic v0.36 (56) and quality checked by FastQC v0.11.5 and MultiQC v1.1 (57). Sequence reads with >75% of the read length in the green zone of the mean quality scores graph on FastQC (>Q28), that have an average read length of >120 bp, and that gave the majority of reads over 140 bp according to the sequence length distribution graph were selected. *De novo* assemblies were generated with the SPAdes assembler v3.12.0 (58), the quality of the assemblies was analyzed using QUAST 4.6.3, and annotation was performed by using Prokka. The quality of assemblies was determined based on the number of contigs with a length of ≥500 bp being less than 500 and the total length of the assembled contigs being similar (within 30%) to the expected genome median from the NCBI genome (34).

### SNP-based phylogenetic analysis.

To perform phylogenetic analysis, 61 recently sequenced strains from Australia and travel-associated strains, as well as 18 international and seven local O1 genome sequences obtained from a public database (NCBI; https://www.ncbi.nlm.nih.gov), were used. Core single nucleotide polymorphisms (SNPs) were determined using Snippy version 4.3.6 (https://github.com/tseemann/snippy) using the seventh-pandemic V. cholerae O1 El Tor N16961 genome as a reference (GenBank accession numbers NZ_CP028827.1 and NZ_CP028828.1). Core SNPs were aligned and used to generate a maximum likelihood tree using FastTree v2.1.10. The distance matrix was constructed with snp-dists v0.6.2. Interactive Tree of Life (iTol) v5.6 was used for the visualization of the phylogenetic tree (https://itol.embl.de/) (59).

### Genomic characterization of NOVC strains from Australia and selected publicly available strains.

All the V. cholerae strains selected for this study were previously identified and characterized using a combination of biochemical, serological, and molecular methods. Furthermore, in this study, whole-genome sequences of all the strains were characterized by using the CholeraFinder (https://cge.food.dtu.dk/services/CholeraeFinder/) database as described in our previous study (34) (online tool CholeraFinder v1.0). This tool uses BLAST as a search engine and was used to determine the presence of the species-specific gene (*ompW*), serogroup-specific genes (*rfbV*-O1, *wbfZ*-O139), biotype-specific genes (*ctxB*, *rstR*, *tcpA*), seventh-pandemic-specific gene (*VC2346*), several virulence genes (*ctxA*, *ctxB*, *zot*, *ace*, *tcpA*, *hlyA*, *stn*, *chxA*, *rtxA*, *ompU*, *toxR*, *mshA*, *makA*, *als*, TTSS, T6SS), and pathogenic islands (VPI-1, VPI-2, VSP-I, VSP-II) with a threshold equal to 95% identity and 60% coverage as previously described (40). For *ctxB* genotyping, the *ctxB* gene sequences were extracted from annotated strains and translated protein sequences were analyzed in Geneious (https://www.geneious.com/) using a multiple-sequence alignment tool, CLUSTALW, (http://www.clustal.org/clustal2/). This was used to determine sequence similarities and uniqueness among *ctxB* protein sequences among strains from Australia.

Antimicrobial resistance genes, mobile genetic element (SXT), and class 1 integron genes were detected using the ResFinder tool (https://cge.food.dtu.dk/services/ResFinder/) for all V. cholerae strains with a threshold equal to 95% identity and 60% coverage as previously described (40).

All the whole-genome-sequenced assemblies (Fasta sequence) were submitted to the PHASTER online web interface (https://phaster.ca/) using default settings to detect the presence of V. cholerae prophages. Results were tabulated from *ctxB* gene-containing strains, and a genome comparison visualizer, Easyfig 2.2.2 (https://mjsull.github.io/Easyfig/), was used to compare and visualize CTX phage regions as described previously (60).

### Data availability.

Raw sequence files and associated metadata have been submitted to the NCBI with BioProject identifier (ID) PRJNA915130.
